# Metabolic response of the Siberian wood frog *Rana amurensis* to extreme hypoxia

**DOI:** 10.1038/s41598-020-71616-4

**Published:** 2020-09-03

**Authors:** Sergei V. Shekhovtsov, Nina A. Bulakhova, Yuri P. Tsentalovich, Ekaterina A. Zelentsova, Lyudmila V. Yanshole, Ekaterina N. Meshcheryakova, Daniil I. Berman

**Affiliations:** 1 Institute of the Biological Problems of the North FEB RAS, Magadan, Russia; 2 Kurchatov Genomic Center, Institute of Cytology and Genetics SB RAS, Novosibirsk, Russia; 3 Tomsk State University, Tomsk, Russia; 4 International Tomography Center SB RAS, Novosibirsk, Russia; 5 Novosibirsk State University, Novosibirsk, Russia

**Keywords:** Metabolomics, Animal physiology, Herpetology

## Abstract

The Siberian wood frog *Rana amurensis* is a recently discovered example of extreme hypoxia tolerance that is able to survive several months without oxygen. We studied metabolomic profiles of heart and liver of *R. amurensis* exposed to 17 days of extreme hypoxia. Without oxygen, the studied tissues experience considerable stress with a drastic decrease of ATP, phosphocreatine, and NAD+ concentrations, and concomitant increase of AMP, creatine, and NADH. Heart and liver switch to different pathways of glycolysis with differential accumulation of lactate, alanine, succinate, as well as 2,3-butanediol (previously not reported for vertebrates as an end product of glycolysis) and depletion of aspartate. We also observed statistically significant changes in concentrations of certain osmolytes and choline-related compounds. Low succinate/fumarate ratio and high glutathione levels indicate adaptations to reoxygenation stress. Our data suggest that maintenance of the ATP/ADP pool is not required for survival of *R. amurensis*, in contrast to anoxia-tolerant turtles.

## Introduction

Anoxia is a huge stress for the majority of vertebrates. Amphibians are considered to be relatively anoxia-intolerant, in contrast to a few turtle species and certain fish species^1–3^: the most resistant species are known to survive anoxia for a few days at most. However, a recent study^4^ proved that the Siberian wood frog *Rana amurensis* can tolerate almost complete anoxia at 2–3 °C (below 0.2 mg/L oxygen, i.e., less than 1.5% of the normal concentration at this temperature) for several months. This is on par with the best vertebrate models from other classes. Moreover, while red-eared slider turtles, the most studied anoxia-tolerant tetrapods, are dormant under anoxia, the Siberian wood frog is able to react to stimuli, e.g., to flee when disturbed. *R. amurensis* is thus a unique model of extreme hypoxia tolerance among terrestrial vertebrates.

The natural range of *R. amurensis* includes Northeastern China and Siberia from the Urals to the coast of the Okhotsk sea; its northern distribution is limited by about 71° N. It overwinters under ice in water bodies. Many of these lakes are shallow (< 3 m) due to thermokarst origin, i.e. resulting from local melting of permafrost. Experimental studies^4^ demonstrated that during the winter oxygen is depleted in overwintering sites till almost complete anoxia. Siberian wood frogs overwinter for 6 to 7 months in different regions under hypoxia until they can leave the lakes in April–early May.

The mechanisms of adaptation to the lack of oxygen are totally unknown for *R. amurensis*. Here we made an attempt to study this phenomenon using ^1^H-NMR-based quantitative metabolomics. This method simultaneously yields concentrations of multiple metabolites^5^. At the present, the detailed quantitative metabolomic composition is known for many human tissues, but for very few animals^6–8^. To the best of our knowledge, this is the first report on the quantitative metabolomic analysis of amphibian tissues. We determined concentrations of over 50 metabolites in heart and liver of *R. amurensis* exposed to 17 days of extreme hypoxia at 2 °C, as well of control specimens kept in normoxia at the same temperature. Our aim was to clarify the metabolic reactions of *R. amurensis* to exposure to extreme hypoxia, including the changes in concentrations of the most important substances involved in energetic processes: glycolysis and the Krebs cycle.

## Results

For the four analyzed types of samples (heart and liver in frogs exposed to extreme hypoxia, as well as in control animals) we quantified a total of 54 metabolites (Table S1). The presence of compounds identified by NMR spectroscopy was confirmed with the use of LC–MS method (Table S1). For each tissue, the measurements were performed for six samples obtained from different individuals.

The comparison of the data obtained for heart and liver of the control animals showed that the concentrations of many metabolites were similar despite the different functions of these organs. The most significant differences were observed for compounds related to the cellular energy generation (creatine, lactate). The levels of compounds which usually play the role of intracellular osmolytes (taurine, *N-*acetyl-histidine, serine-phosphoethanolamine, glycerophosphocholine) in the heart were also higher.

**Figure 1:**
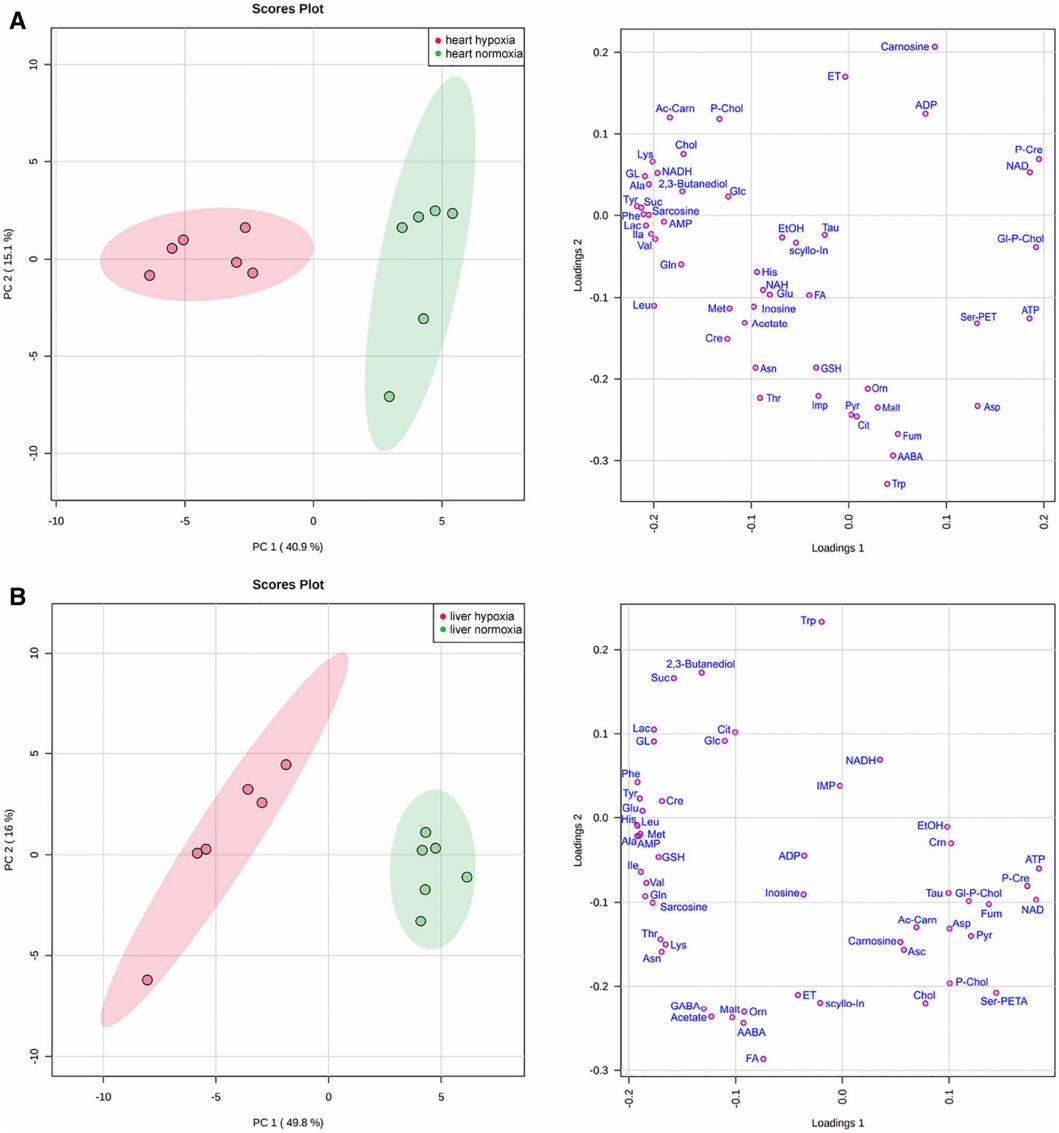
Scores (left) and loadings (right) plots of principal component analysis (PCA) of *R. amurensis* metabolomics profiles*.* (**A**) heart; (**B**) liver. The data are range scaled. Colored ovals indicate 95% confidence regions: red, extreme hypoxia; green, control. Variance explained by the first (PC1) and second (PC2) principal components are indicated on the axes of the scores plots.

The principle component analysis (PCA; Fig. 1A,B, left panels) showed that the normoxic and hypoxic samples were well separated for both heart and liver, and the data spread within each group is relatively small. Noteworthy, the majority of metabolites were situated at the periphery of the loadings plots (Fig. 1A,B, right panels). The hypoxia-induced changes in the data sets for metabolite concentrations can also be seen on the Volcano plot (Fig. S1). It demonstrated that the highest and statistically significant increase in both organs was found for glycerol, 2,3-butanediol, lactate, alanine, phenylalanine, and some other amino acids, and the decrease, for ATP, aspartate, and phosphocreatine.

We detected some differences between the sexes even given low sample sizes (3 males: 3 females). Female hearts contained less histidine in normoxia and less phenylalanine in extreme hypoxia. Female normoxic liver contained more asparagine, aspartate, leucine, *N-*acetylcarnitine, and less creatine. Under extreme hypoxia, female liver had more alanine, glutamine, methionine, and sarcosine. Hypoxic female livers also contained more scyllo-inositol and less pyruvate and tryptophan, but the values for these substances were very low, and thus the detected differences are probably not reliable, although statistically significant.

Differences between males and females were found to be minor both in control and extreme hypoxia.

### Small molecules

**Figure 2:**
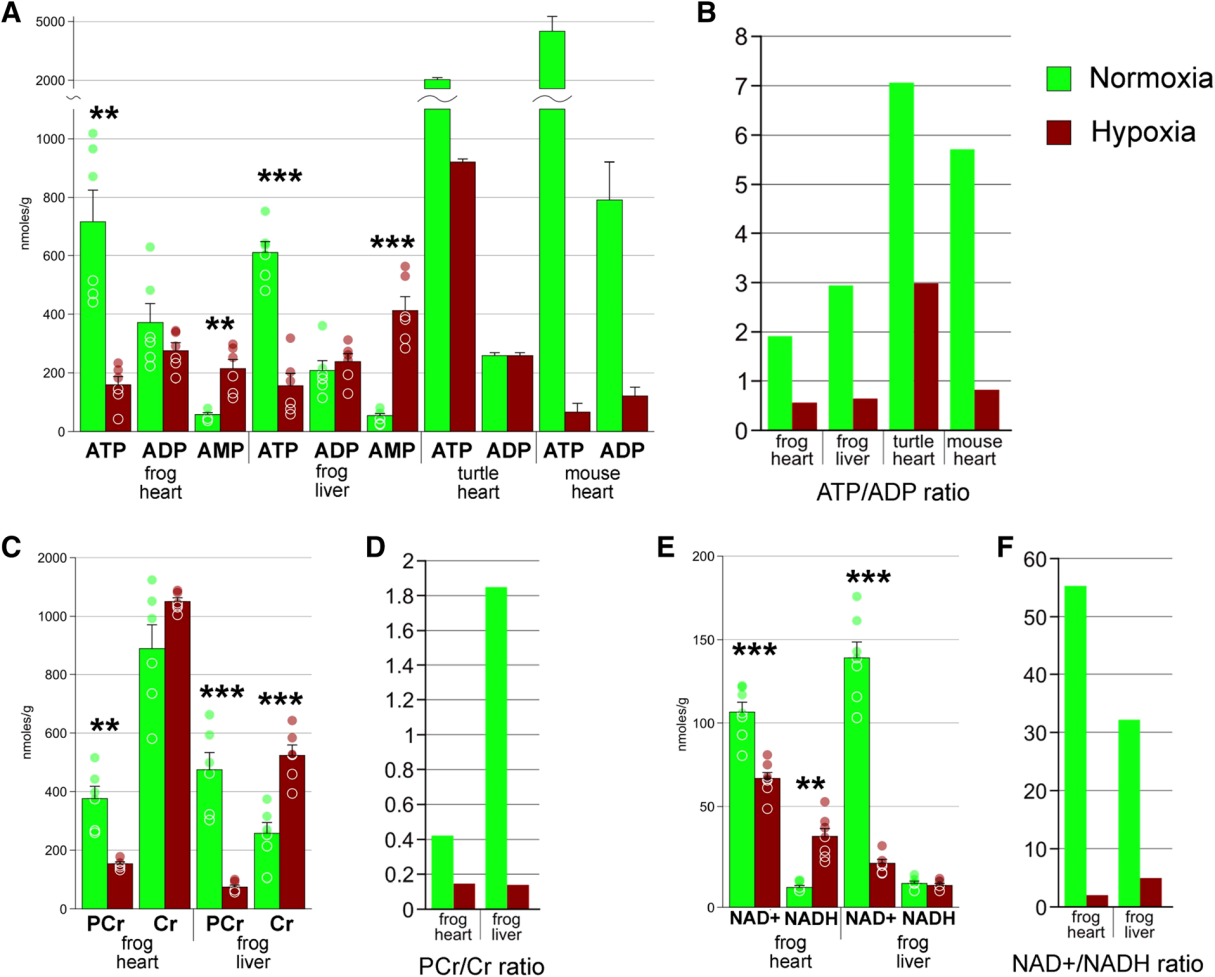
Concentrations of small energy molecules. (**A**) absolute concentrations of ATP, ADP, and AMP for frog heart and liver, as well as for the turtle *Trachemys scripta* in normoxia and 9 days of extreme hypoxia, and for mouse heart under 90 min of ischemia (data from Brungaard et al. 2019). (**B**) average ATP/ADP ratio for the data presented in (**A**). (**C**) absolute concentrations of phosphocreatine (PCr) and creatine (Cr). (**D**) PCr/Cr. (**E**) concentrations of NAD + and NADH. (**F**) NAD + /NADH ratio. Green columns, normoxia; red, hypoxia; *, Welch's test *p* < 0.05; **, *p* < 0.01; ***, *p* < 0.001; circles, individual data points; bar, SE.

As seen in Fig. 2A, under extreme hypoxia ATP content was about 4.5 times as low in heart and 3.5 times as low in liver compared to normoxia. AMP content increased approximately 3.5 and 8 times, respectively. ADP levels did not change in response to extreme hypoxia.

Similar changes were observed for the phosphocreatine/creatine (PCr/Cr) pair. PCr concentration decreased 2.4 times in heart and 6.3 times in liver, while Cr decreased by 1.2 and 2 times, respectively (Fig. 2C).

Changes in NAD+/NADH concentrations were extremely pronounced under extreme hypoxia (Fig. 2E). NAD+ content was 1.5 times lower in heart and 8.5 lower in liver in hypoxia compared to normoxia. NADH content increased by 17 times in heart but remained at the same level in liver.

We also detected inosine, inosinate, and creatinine. Their levels remained constant throughout the studied samples (not shown). Other nucleobases, most importantly the products of purine degradation, xanthine and hypoxanthine, were not detected.

### Glycolysis and the Krebs cycle

**Figure 3:**
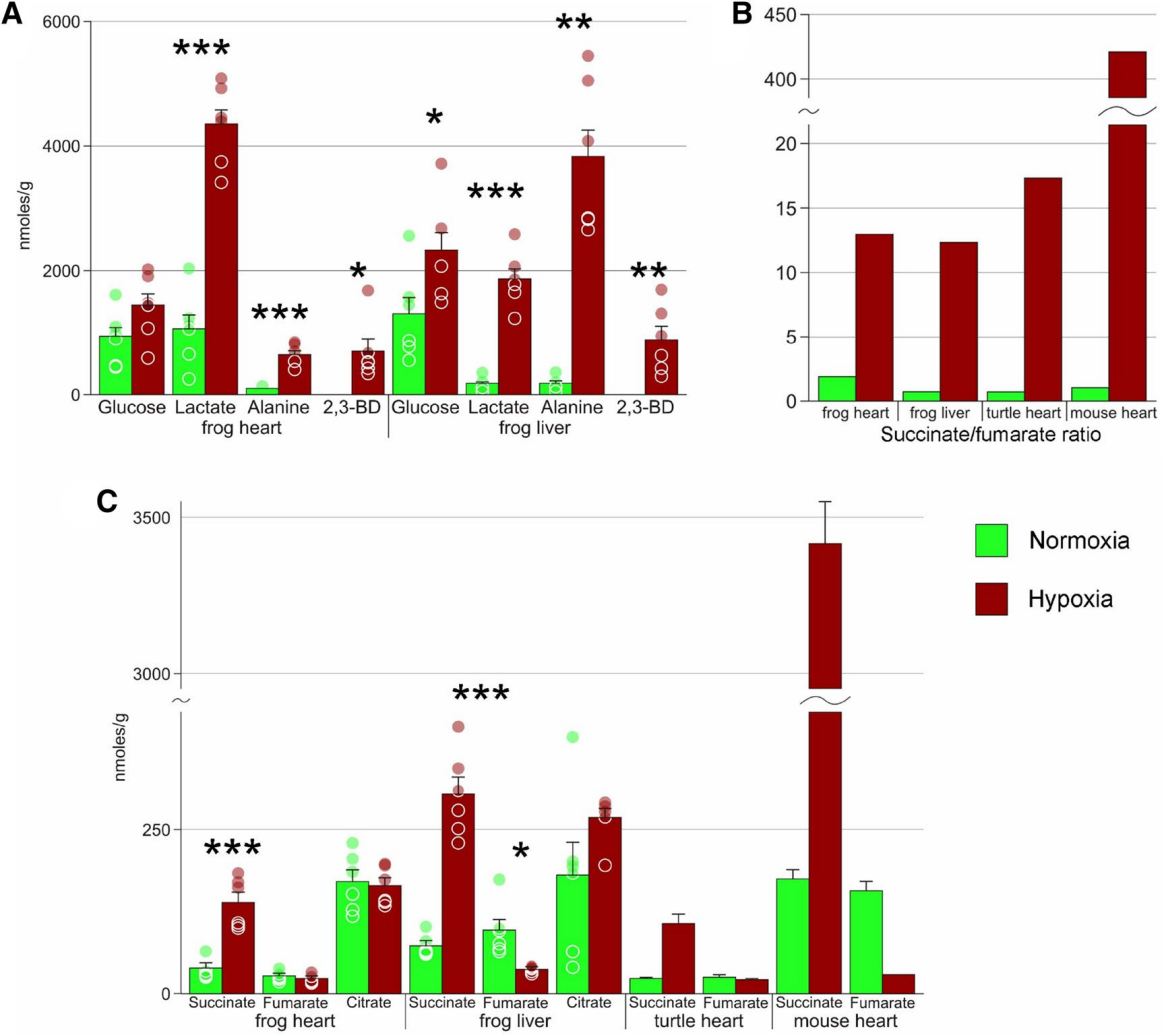
Components of glycolysis and the Krebs cycle. (**A**) absolute concentrations of glycose and glycolysis end products in normoxia and extreme hypoxia; data for the snapping turtle and for mouse taken from Brungaard et al. (2019). (**B**) succinate to fumarate ratio for the data presented in (**A**). (**C**) concentrations of Krebs cycle intermediates in normoxia and extreme hypoxia; data for the snapping turtle and for mouse taken from Brungaard et al. (2019). Green columns, normoxia; red, hypoxia; *, Welch's test *p *< 0.05; **, *p *< 0.01; ***, *p *< 0.001; circles, individual data points; bar, SE.

Under extreme hypoxia, average glucose concentration in liver increased 1.8-fold (Fig. 3A). We also observed 1.5-fold increase in glucose concentration in heart, but these differences were not statistically significant due to high variation. It should be noted that free glucose concentration in the studied organs was high both in hypoxia and normoxia.

Lactate concentration increased by almost six times in heart, and ten times, in liver under extreme hypoxia (Fig. 3A). Two other compounds acting as end products were alanine and 2,3-butanediol. Average alanine concentrations rose sixfold in heart and tenfold in liver. 2,3-butanediol was below the detectable level in normoxia but was accumulated in significant amounts in hypoxic organs. Pyruvate and ethanol were present in negligible quantities (not shown). No traces of any glycolysis intermediates were found.

We found three members of the Krebs cycle: succinate, fumarate, and citrate (Fig. 3C). Succinate content increased almost fivefold both in heart and in liver. Fumarate concentration decreased 3.5-fold in liver, but remained unchanged in heart. Citrate content showed no change in response to extreme hypoxia in either organ.

### Amino acids

**Figure 4:**
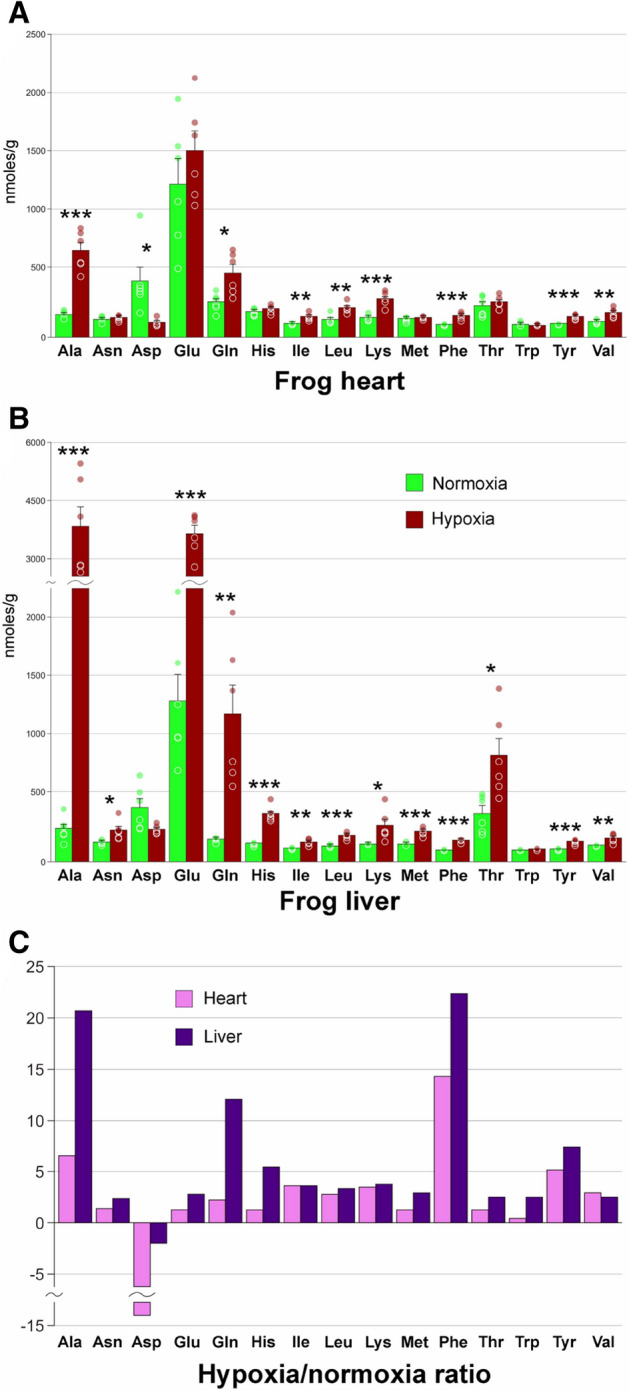
Proteinogenic amino acids. (**A**) absolute concentrations of the 15 detected amino acids in frog heart. Green columns, normoxia; red, extreme hypoxia; *, Welch's test *p* < 0.05; **, *p* < 0.01; ***, *p* < 0.001; circles, individual data points; bar, SE. (**B**) absolute concentrations of amino acids in frog liver. Abbreviations are the same as in (**A**). (**C**) Ratio of average amino acid concentrations in hypoxia to normoxia (for aspartate, the negative value of normoxia to hypoxia ratio). Pink, heart; purple, liver.

We assessed concentrations of 15 proteinogenic amino acids (Fig. 4A). Glycine was determined for control frogs, but in the spectra of hypoxic specimens it could not be quantified due to the overlap with the strong glycerol signal. The remaining four amino acids were not found neither in normoxia nor in hypoxia. All amino acids except for aspartate were more abundant under extreme hypoxia compared to normoxia, and these differences were statistically significant in most of the cases (Fig. 4A,B). Differences in amino acid concentrations were more pronounced in liver.

### Other metabolites

**Figure 5:**
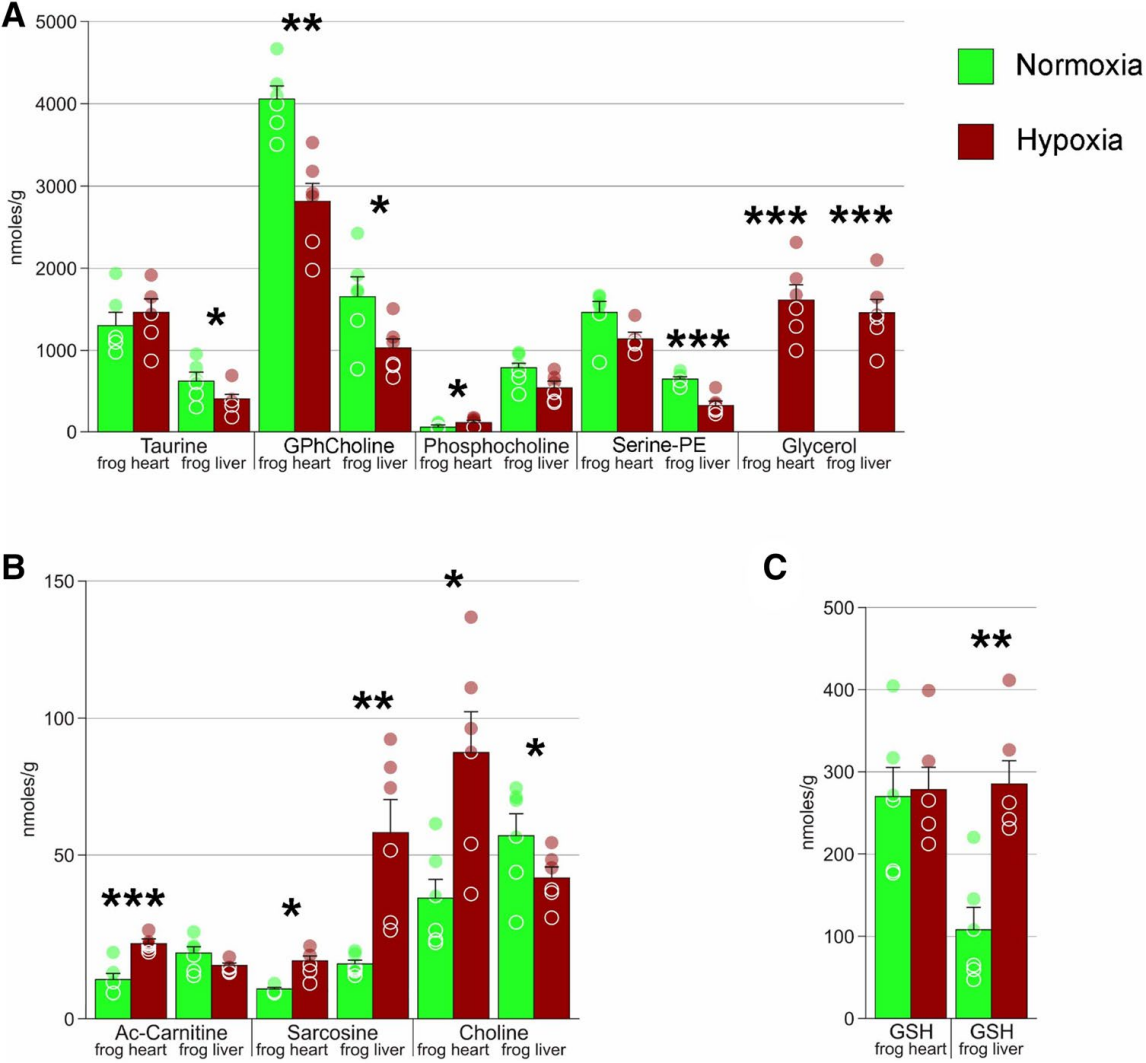
Concentrations of various metabolites in *R. amurensis* heart and liver. (**A**) GPhCholine, glycerophosphocholine; Serine-PE, serine phosphoethanolamine. (**B**): Ac-Carnitine, *N-*acetylcarnitine. (**C**) GSH, gluthatione. Green columns, normoxia; red, hypoxia; *, Welch's test *p* < 0.05; **, *p* < 0.01; ***, *p* < 0.001; circles, individual data points; bar, SE.

We found statistically significant changes in levels of many biologically important compounds. Significant levels of glycerol were accumulated in hypoxic organs while totally absent in normoxia (Fig. 5A). Sarcosine concentration increased in both heart and liver (Fig. 5B), while glycerophosphocholine and serine-phosphoethanolamine decreased. Changes in the levels of other compounds were organ-specific: under extreme hypoxia, there was more phosphocholine in liver but less in heart. Taurine concentration decreased in liver but not in heart. We also observed significant increase in *N-*acetylcarnitine and choline in heart, although concentrations of the former were minor both in hypoxia and normoxia.

Glutathione level increased 2.5-fold in liver under extreme hypoxia (Fig. 5C). In heart, the concentration of this compound in control frogs was high, similar to that in hypoxic liver, and did not change in response to extreme hypoxia.

Concentrations of several metabolites were unaffected by extreme hypoxia. These include *N-*Ac-histidine, ornithine, ergothioneine, AABA, GABA, acetate, ascorbate, formate, scyllo-inositol, maltose, creatinine, inosinate, and inosine.

## Discussion

### Glycolytic pathways

Under extreme hypoxia, animals have to switch from oxidative phosphorylation to glycolysis. During glycolysis, glucose is processed via a series of intermediates to pyruvate with ATP yield. Pyruvate then has to be converted into various end products in order to restore NAD+. The most widespread of those are lactate, alanine, and ethanol, although many less common ones are known to exist^9^. Two pathways predominate in tetrapods: pyruvate is either directly converted to lactate, or reacts with glutamate in a transamination reaction yielding alanine and α-ketoglutarate. Glutamate concentration is then restored by the malate-aspartate shuttle reactions, while alanine is accumulated.

Both lactate and alanine are known to accumulate in various organs of the anoxia-tolerant turtles *Chrysemys picta* and *Trachemys scripta*^10,11^, as well as in the embryos of the annual killifish *Austrofundulus limnaeus*^12^. The naked mole-rat *Heterocephalus glaber*, a mammalian model of hypoxia tolerance, accumulates lactate at 5% oxygen concentrations^13^. The crucian carp *Carassius carassius* demonstrates elevated levels of lactate under anoxia^14^, despite the fact that ethanol is usually considered as its anaerobic end product^15^.

We found that both lactate and alanine accumulate in *R. amurensis* in response to extreme hypoxia. As shown in Fig. 3A, pyruvate conversion pathways were shifted towards lactate in heart and towards alanine, in liver.

We should note here that high alanine concentrations might also be the result of the amino acid degradation and the concomitant accumulation of the substances used to store and transport ammonium ions (glutamate, glutamine, and alanine, discussed below in the section “Amino acids”).

There was one more compound, 2,3-butanediol (2,3-BD), that was absent in normoxia but present in significant concentrations in extreme hypoxia (Fig. 3A). It is one of the end products of bacterial glycolysis, and is synthesized from pyruvate via acetoin by means of several alternative pathways^16^. In humans, 2,3-BD was detected as one of the products of ethanol degradation^17–19^, as well as in patients with certain forms of cancer^20^. Moreover, 2,3-BD is a marker of cardiac ischemia in humans, and is accumulated alongside lactate in ischemic pig hearts^21,22^. These facts suggest that 2,3-BD might be one of the end products of glycolysis alongside with lactate and alanine in *R. amurensis*, as well as in other vertebrates. One of its presumable advantages is that it is a neutral metabolite in contrast to lactate^16^. However, synthesis of 2,3-BD in bacteria has lower efficiency of NADH to NAD+ conversion compared to other glycolysis pathways, since only one molecule of 2,3-BD is formed from two molecules of pyruvate. Surprisingly, the pathways of 2,3-BD biosynthesis in animals are unknown; the findings that 2,3-BD in humans is formed after ethanol intake implies other reaction pathways.

Another putative end product of glycolysis is glycerol, also present in high amounts in extreme hypoxia but absent in normoxia (Fig. 5A). Glycerol is a well-known cryoprotectant in amphibians^23–26^. However, its presence in our sample could not be explained by adaptation to low temperatures, since control frogs were kept at the same temperature but did not accumulate any glycerol. The phenomenon of glycerol accumulation in response to hypoxia was never reported for animals, but is known to occur in yeast^27,28^ and trichomonads^29^. Under anoxia, *Saccharomyces cerevisiae* produces large amounts of glycerol in addition to the main product of glycolysis (ethanol), presumably as a means to restore NAD+ levels^28^. If this is the case for *R. amurensis*, glycerol may also be considered an end product of glycolysis. However, there is an alternative explanation: high glycerol levels may be the result of glycerophosphocholine hydrolysis. Concentrations of glycerophosphocholine were found to decrease both in heart and liver (Fig. 5A); however, no concomitant changes in the quantities of phosphocholine or choline were observed.

Increased concentrations of glucose in liver indicate activation of glycogenolysis. In heart, changes in glucose concentration were not significant, which probably reflects the balance of increased glucose delivery and consumption. We should note that glucose concentrations in normoxic frogs were also high, probably as a response to low temperatures. It is hypothesized that other sugars may play an important role in anoxia response. For example, fructose is an important energy source in the naked mole-rat under hypoxia^13^. However, we found only a relatively minor amounts of maltose in equal amount in extreme hypoxia and normoxia.

### Rearrangement of energy pathways

The absence of oxygen leads to the termination of the electron transfer chain and to dramatic rearrangements of the Krebs cycle. A universal feature here is the accumulation of succinate, which was shown to be due to the reversal of the Krebs cycle^30^. Without oxygen, succinate dehydrogenase acts in reverse, forming succinate from fumarate. The malate-aspartate shuttle and the purine nucleotide pathway contribute to this process in ischemic mammalian tissues^30–32^. Succinate accumulation was also observed in various hypoxia-tolerant vertebrates^10–13^. We demonstrated that this phenomenon holds true for *R. amurensis* as well (Fig. 2C).

The obtained data (Fig. 2A–F) indicate that extreme hypoxia dramatically reduces available energy reserves. This is demonstrated by extreme changes in ATP/ADP and PCr/Cr ratios. However, the absence of xanthine and hypoxanthine, the products of purine degradation that are observed in high concentrations during ischemia in mammals^11^ suggests that this stress is reversible.

What metabolic features are associated with hypoxia tolerance? Brungaard^11^ suggested that two major patterns are observed in red-eared slider turtles, i.e., (1) high ATP/ADP ratio (relative to intolerant species) and no signs of their degradation to AMP, xanthine, and hypoxanthine; (2) low succinate/fumarate ratio. For the Siberian wood frog, rule no. 2 holds true (Fig. 3B). However, ATP/ADP ratio, as well as the absolute quantities of ATP in extreme hypoxia and even in normoxia are low, similar to those in mammalian heart under ischemia (Fig. 1A). Moreover, degradation to AMP (but not xanthine or hypoxanthine) is observed. We can state, however, that such changes are far from lethal: the samples were taken from frogs exposed to 17-day extreme hypoxia, while we know that they could survive this state for further three to 5 months^4^. Low ATP levels compared to turtles may be accounted for by different systematic position of these model organisms. We can thus conclude that low ATP content does not impede hypoxia survival.

### Amino acids

Two amino acids are believed to play an important role in hypoxia: alanine, the end product of glycolysis, and aspartate that is depleted by the malate-aspartate shuttle. As seen in Fig. 4 and Table S1, alanine was among the most abundant and the most affected by extreme hypoxia both in liver and heart (changes in phenylalanine concentrations were higher, but the background content was low). Aspartate was the only amino acid that decreased in response to extreme hypoxia (Fig. 4). One can also see that changes in aspartate levels were much higher in heart compared to liver, in agreement with higher alanine concentrations in that tissue.

Increased concentrations of all amino acids except for aspartate can be explained by inhibition of ribosomal synthesis in order to save energy. Translation arrest is observed in red-eared slider turtles in response to anoxia^33,34^. However, the increase of free amino acids is not a universal response in anoxia-tolerant animals. Increased concentrations of all amino acids are observed only in embryos of the annual killifish *Austrofundulus limnaeus*^12^. Red-eared slider turtles demonstrate a mixed response whereby five amino acids including alanine are upregulated, and the rest, downregulated^11^. A similarly ambiguous response is also characteristic for *Carassius carassius*^14^.

However, the obtained data imply that glutamine and glutamate must have a more direct role in hypoxia response (Fig. 4). Moreover, increases in concentrations of these amino acids are much more pronounced in liver compared to heart. One possible option is that these amino acids accumulate as the result of protein and amino acid catabolism. Deamination of amino acids yields glutamate that is used to hold excess ammonium^35^. It is normally converted back to α-ketoglutarate, but this process should be impeded under the paucity of NAD+ during hypoxia. In some organs such as muscle, glutamate is then converted to glutamine or alanine that are transported to the liver, where they are catabolized^35^. Under extreme hypoxia, their processing may be impeded, so this may be the reason we observe high quantities of these amino acids in liver but not in heart.

### Other molecules

A number of compounds changed their concentrations in response to extreme hypoxia. Sarcosine (upregulated in both tissues) and taurine (downregulated in liver) are usually regarded as osmolytes^9^, but may also have multiple other functions^36–38^. Changes in concentrations of choline, phosphocholine, glycerophosphocholine, and serine-phosphoethanolamine are probably associated with changes in phospholipid metabolism^39^. Moreover, glycerphosphocholine is an osmolytes^9^, while choline is the precursor of acetylcholine, an important neuromediator. Taurine is also considered a neuroregulatory substance; increased taurine concentrations were also observed in anoxic carp muscle^14^. Multiple functions of the aforementioned substances make it hard to state which role they play in hypoxia response.

Reoxygenation stress is an important factor in hypoxia-related damage. Adaptation to this stress is thus a vital part of hypoxia response. In accordance with this, concentrations of glutathione increased 2.5-fold in liver under extreme hypoxia (Fig. 5C). Concentrations of glutathione in the heart were high both in hypoxic and control frogs. Heart is one of the most important and sensitive organs, so high intrinsic levels might reflect preadaptation to reoxygenation stress. Similarly high concentrations of glutathione are observed in red-eared slider turtles^40^.

## Conclusions

In this study we demonstrated that extreme hypoxia causes large-scale rearrangements in phospholipid and nitrogen metabolism, protein degradation, accumulation of free amino acids and osmolytes in the Siberian wood frog *R. amurensis*. The studied tissues have different modes of glycolysis with a variety of the accumulated end products. The Krebs cycle is halted with the accumulation of succinate, similar to other anoxia-tolerant tetrapods. Our data suggest that maintenance of ATP/ADP pool is not necessary for frog survival.

## Materials and methods

### Animals

*R. amurensis* individuals were collected in September 2019 in the environs of Lesopilnoye village, Khabarovsk Krai (46° N, 134° E). Frogs were collected using approved methods under appropriate permits issued by cognizant governmental agencies (№ 001/04-19).

### Animal care

The collected frogs were distributed by 5–7 individuals into 10 L containers filled with water (oxygen level 7–8 mg/L) and kept for 2 days at 14–15 °C, for 4 days at 8, 4, and 2–3 °C. In nature, the Siberian frogs do not feed in winter, so they were not fed during the experiment. Twelve individuals (six males and six females; 24.1 ± 0.8 g; 21.9–27.1 g) were randomly allotted among the control and experimental groups. Six individuals (three males and three females) were exposed to extreme hypoxia and other six remained in the control group. The control group was kept in open containers in water with an oxygen level no less than 5–7 mg/L, at 2–3 °C.

All the procedures were carried out in accordance with the International Guiding Principles for Biomedical Research Involving Animals (Council for International Organizations of Medical Sciences, 1985) and the European Union Directive 2010/63/EU on the protection of animals used for scientific purposes. Experimental protocols were approved by the Bioethics Committee of Institute of Biological Problems of the North.

### Preparation of water for experiments

We followed the protocol of water preparation described in^4^: tap water was kept in open containers for 2 days, then poured into 6.3 L containers with narrow necks (38 mm in diameter) and with hermetical screw caps, and cooled down to 2–3 °C. The frogs were placed into the containers, the lids were tightened, the absence of an air bubble was checked, and the containers were placed into cooling chambers with a temperature of 2–3 °C.

The dissolved oxygen content was measured by a HACH HQ30D Flexi digital single-channel multiparameter device with a luminescent LDO101 sensor^41^; the accuracy of the device was 0.1 mg/L. The probe was calibrated before the experiment. The oxygen level was measured daily until it reached 0.2 mg/L. Measuring dissolved oxygen in each container took at most 3–4 min; the water replaced by the probe was replenished with previously prepared water of the same oxygen concentration. The tightness of the lids was regularly checked after measuring. The state of frogs (motor activity, level of behavioral frustration) was monitored every 24 h by visual inspection through the transparent walls of the containers.

These closed (experimental group) or open (control) containers were sent by plane to the Institute of Cytology and Genetics SB RAS (Novosibirsk, Russia) in thermostated boxes at 2–3 °C. After three days (a total of 17 days in extreme hypoxia) the airtight containers were opened, oxygen content was measured as described above, and the animals were slaughtered. Tissues were quickly (20–30 s) excised and frozen in liquid nitrogen for extraction of metabolites.

### Chemicals

Chloroform and methanol were purchased from PanReac (Spain). D_2_O 99.9% was purchased from Armar Chemicals (Switzerland). All other chemicals were purchased from Sigma-Aldrich (USA). H_2_O was deionized using Ultra Clear UV plus water system (SG Water, Germany) to the quality of 18.2 MΩ.

### Frog heart and liver preparation

Each sample of the frog tissue was weighted prior to homogenization: the typical heart sample weight was 70 mg and the typical liver sample weight was 175 mg. The tissue was placed in a glass vial and homogenized with a TissueRuptor II homogenizer (Qiagen, Netherlands) in 1,600 µL of cold (− 20 °C) MeOH, and then 800 µL of water and 1,600 µL of cold chloroform were added^42^. The mixture was shaken well in a shaker for 20 min and left at − 20 °C for 30 min. Then the mixture was centrifuged at 16,100*g*, + 4 °C for 30 min, yielding two immiscible liquid layers separated by a protein layer. The upper aqueous layer (MeOH-H_2_O) was collected and lyophilized.

### NMR measurements

The extracts for NMR measurements were re-dissolved in 600 μL of D_2_O containing 6 × 10^−6^ M sodium 4,4-dimethyl-4-silapentane-1-sulfonic acid (DSS) as an internal standard and 20 mM deuterated phosphate buffer to maintain pH 7.2.

The ^1^H-NMR measurements were carried out in the Center of Collective Use «Mass spectrometric investigations» SB RAS on a NMR spectrometer AVANCE III HD 700 MHz (Bruker BioSpin, Germany) equipped with a 16.44 T Ascend cryomagnet as described in^43^. The proton NMR spectra for each sample were obtained with 64 accumulations. Temperature of the sample during the data acquisition was kept at 25 °C, the detection pulse was 90°, and the repetition time between scans was 12 s. Low power radiation at the water resonance frequency was applied prior to acquisition to presaturate the water signal. The concentrations of metabolites in the samples were determined by the peak area integration respectively to the internal standard DSS.

### LC–MS measurements

In this work, LC–MS measurements were performed only for the confirmation of data obtained by the NMR method. The LC separation was performed on a UltiMate 3000RS chromatograph (Dionex, Germany) using a hydrophilic interaction liquid chromatography (HILIC) method on a TSKgel Amide-80 h (Tosoh Bioscience, Germany) column (4.6 × 250 mm, 5 μm) as described earlier^43^. The MS detection in both positive and negative modes was performed with the use of an ESI-q-TOF high-resolution hybrid mass spectrometer maXis 4G (Bruker Daltonics, Germany) connected to the chromatograph. The data processing was performed using software *peakonly*^44^ with the following parameters: m/z window, ± 0.005 Da; minimal length of ROI, 15 points; minimal peak length, 8 points; maximal number of zero points in a row, 3.

### Data analysis

The principal component analysis (PCA) was performed on a MetaboAnalyst 4.0 web-platform (www.metaboanalyst.ca)^45^. PCA scores, loading plots and Volcano plots were constructed with the range data scaling to normalize the contributions of all metabolites^45^.

^1^H-NMR spectra of protein-free lipid-free extracts from heart and liver of *R. amurensis* were obtained for frogs exposed to extreme hypoxia (n = 6) and for control animals (n = 6). The signal assignment in the spectra was performed according to the metabolite NMR spectra available in literature and in our in-house library^46–48^. In questionable cases, signal attribution was confirmed by spiking the extract with commercial standard compounds. Typical NMR spectra of the frog heart and liver are shown in Figs. S2–S4, and some examples of the spiking spectra are presented in Figs. S5–S7. Metabolite concentrations in extracts were measured by the NMR signal integration relatively to the internal standard DSS followed by the calculation of metabolite concentrations in a tissue (in nmol per gram of the tissue wet weight). Signals from some metabolites were strongly overlapped by other signals or too weak for the reliable integration. Despite the positive identification, these compounds were excluded from the analysis, and in this work only the compounds with reliable quantification are present.

Differences between the samples were assessed using two-tailed Welch’s test implemented in MS Excel.

## Supplementary information

Supplementary Tables.

Supplementary Figures.

## Data Availability

All data generated in this study are included in this published article as Supplementary Information files.
