# Supplementary material for: Metabolic response of the Siberian wood frog Rana amurensis to extreme hypoxia
Source: Sci Rep. 2020 Sep 3;10:14604. doi: 10.1038/s41598-020-71616-4 (PMC7471963; doi:10.1038/s41598-020-71616-4)
Supplement: Supplementary file 2 — Supplementary Figures. [file 41598_2020_71616_MOESM2_ESM.docx]

**Metabolic response of the Siberian wood frog *Rana amurensis* to extreme hypoxia**

Sergei V. Shekhovtsov^1,2,*^, Nina A. Bulakhova^1,3^, Yuri P. Tsentalovich^4^, Ekaterina A. Zelentsova^4,5^, Lyudmila V. Yanshole^4^, Ekaterina N. Meshcheryakova^1^, Daniil I. Berman^1^

^1^ Institute of the Biological Problems of the North FEB RAS, Magadan, Russia

^2^ Institute of Cytology and Genetics SB RAS, Novosibirsk, Russia

^3^ Tomsk State University, Tomsk, Russia

^4^ International Tomography Center SB RAS, Novosibirsk, Russia

^5^ Novosibirsk State University, Novosibirsk, Russia

* e-mail: shekhovtsov@bionet.nsc.ru

**Supplementary Figures**


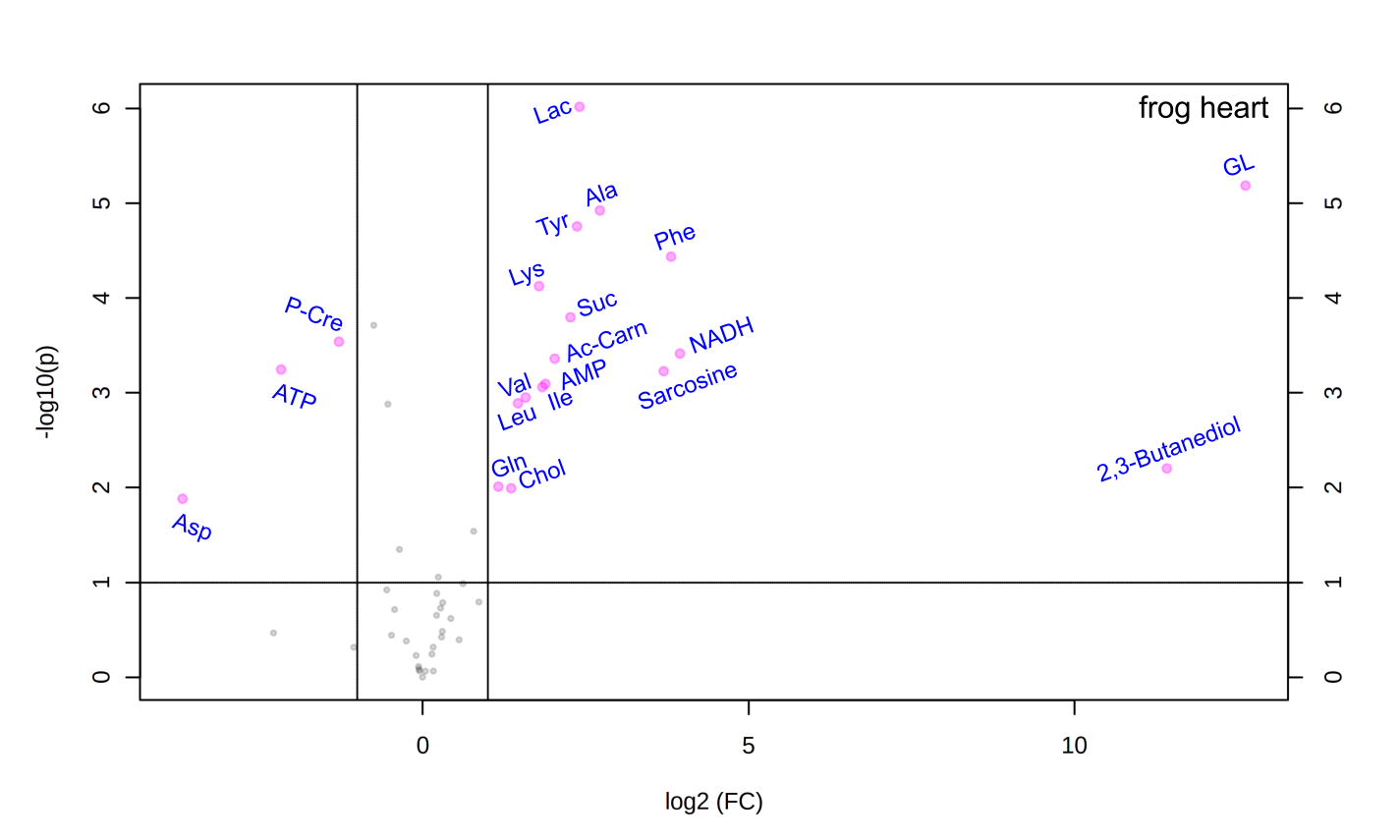


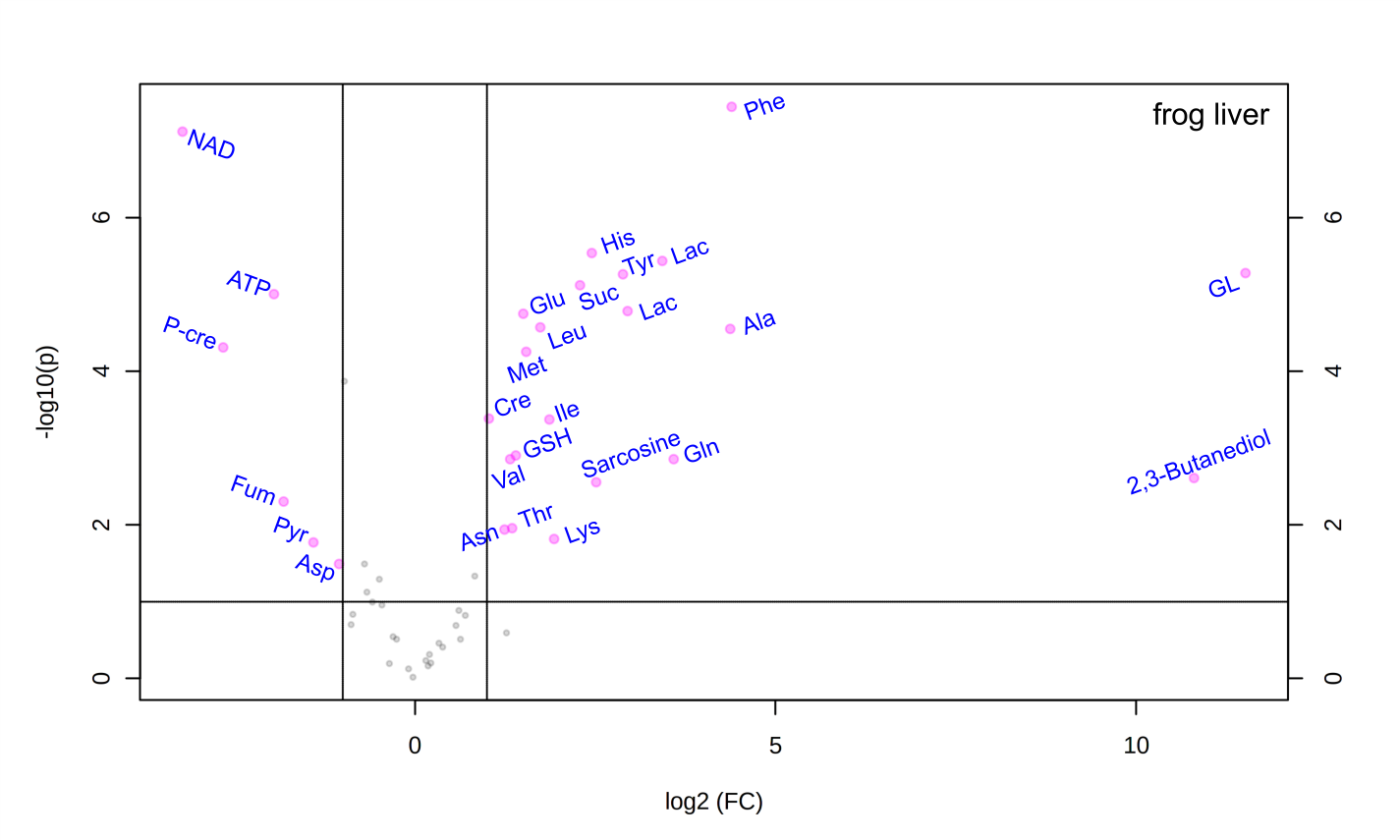


Figure S1. Volcano plots for metabolomic data obtained for the frog heart (upper graph) and liver (lower graph) under normoxia and extreme hypoxia. The metabolites with the highest and statistically significant difference between normoxia and hypoxia are pink colored and annotated.

Figure S2. Aliphatic region (0.85-3.07 ppm) of NMR spectra of protein-free lipid-free extract from the frog liver and heart under normal conditions and after 17 days of extreme hypoxia.

Figure S3. Middle region (3.07-4.67 ppm) of NMR spectra of protein-free lipid-free extract from the frog liver and heart under normal conditions and after 17 days of extreme hypoxia.

Figure S4. Aromatic region (5.17-9.4 ppm) of NMR spectra of protein-free lipid-free extract from the frog liver and heart under normal conditions and after 17 days of extreme hypoxia.

Figure S5. Spiking of liver sample with AMP.

Figure S6. Spiking of liver sample with Gln.

Figure S7. Spiking of liver sample with Leu.
